# Online Doctor Recommendation with Convolutional Neural Network and Sparse Inputs

**DOI:** 10.1155/2020/8826557

**Published:** 2020-10-15

**Authors:** Yongjie Yan, Guang Yu, Xiangbin Yan

**Affiliations:** ^1^School of Management, Harbin Institute of Technology, Harbin 150001, China; ^2^School of Mathematics and Computer Science, Jiangxi Science & Technology Normal University, Nanchang 330038, China; ^3^School of Economics and Management, University of Science and Technology Beijing, Beijing 100083, China

## Abstract

The recommendation system in the online medical consultation website is a system to assist patients to find appropriate doctors. Based on the analysis of the current situation of the development of an online medical community (Haodf.com) in China, this paper puts forward recommendation suggestions of finding the right hospital and doctor to promote the rapid integration of Internet technology and traditional medical services. A new recommendation model called Probabilistic Matrix Factorization integrated with Convolutional Neural Network (PMF-CNN) is proposed in the paper. Doctors' data in Haodf.com were used to evaluate the performance of our system. The model improves the performance of medical consultation recommendations by fusing review text and doctor information based on CNN (Convolutional Neural Network). Specifically, CNN is used to learn the feature representation of the review text and the doctors' information. Furthermore, the extended matrix factorization model is exploited to fuse the review information feature and the initial value of the doctors' information for recommendation. As is shown in the experimental results on Haodf.com datasets, the proposed PMF-CNN achieves better recommendation performances than the other state-of-the-art recommendation algorithms. And the recommendation system in an online medical website improves the utilization efficiency of doctors and the balance of public health resources allocation.

## 1. Introduction

The online medical consultation website is a new type of public health platform formed by the combination of Internet information technology and the medical service industry. There are more and more medical consultation platforms in Chinese online medical website, and people can complete their diagnosis of diseases without leaving their homes. These online medical consultation websites are especially helpful to remote mountainous areas and rural areas that lack higher levels of medical conditions. All kinds of online medical and health services also alleviate the lack of medical resources in certain areas and the imbalance of regional distribution in the country [1]. The analysis of healthcare services based on social media platforms, online doctor reviews, and web-based medical consultation in China are given in [2–4]. However, there are still many problems. For example, the data of each online medical platform in the website are not interoperable, the quality of the platform doctors is uneven, the questions cannot be answered within a limited time, and the condition could be easily misdiagnosed according to the one-sided description.

At present, recommendation algorithms can be generally divided into the following three categories [5, 6], content-based recommendation [7–9], collaborative filtering-based recommendation [10–13], and hybrid recommendation [14–17].

Convolutional Neural Network (CNN) uses layers with convolutional filters to apply local features. CNN was originally invented for computer vision, but, in recent years, lots of studies show that the CNN model has a good effect on recommendation systems and has achieved good results in semantic analysis, search and retrieval, sentence modeling, and other recommendation tasks. Oord et al. [18] directly use CNN to learn effective representations of songs in a content-based recommendation framework. Kalchblenner et al. [19] described a dynamic CNN, which uses a dynamic *k*-max pool operator as a nonlinear subsampling function. Their experiments show that the model can achieve good accuracy without external features or other resources. Kim [20] proposes a simple CNN, which uses a single convolution layer and a max pool layer to achieve similar results. He also proved by experience that, using his multichannel model, he achieved 47.4% and 88.1% accuracy in the 5-tag movie comment emotion classification task (sst-1 dataset) and binary classification task (sst-2 dataset), respectively. CNN based relational extraction system is also shown in the following papers [21–23].

The static pretrained word vectors are trained by Mikolov et al. [24, 25] on 100 billion words of Google News. Cicero and Gatti [26] introduced chars CNN by combining word-level embedding and character level embedding as the input of CNN to extract sentence level representation. In the stage of network training, both character level embedding and character level embedding must be trained. They also prove that the structure of feedforward neural network can be effective in sentence sentiment analysis. Lin et al. [27] proposed a real-time and Continua-based Care Guideline Recommendation System (Cagurs) using mobile device platforms. The key idea of Neural Collaborative Filtering (NCF) [28] is to combine MF and MLP with dual-channel structure and learn the user-item interaction using neural networks based on framework for making recommendations. Ma et al. [29] introduce a new group sparse autoencoders algorithm and a new group sparse CNN, which naturally learns the representation of the problem by embedding group sparse self-coding in the traditional CNN.

A lot of research has been carried out on the problem of recommendation, but the existing recommendation algorithms have the following problems:

1. Most recommendation works associate review information with side information to reduce data sparsity; however, review information has not been fully utilized in related research work. Most of the work of recommendation system using review information focuses on the topic mining of review information using the Latent Dirichlet Allocation (LDA) [30]. However, the model usually uses the word bag model to deal with review information, ignoring the semantic context information of review information. Moreover, when the data is too sparse, the latent feature representation of LDA model learning may not be very effective, and the performance is not satisfactory [31–33].
2. Most researches on recommendation systems based on machine learning use matrix decomposition technology to recommend items [34]. The method based on the matrix decomposition model is very sensitive to the initialization of the latent feature matrix of users and users' interest points [35]. However, most of the recommendation work based on matrix decomposition avoids or ignores this problem and uses very simple methods (such as random or zero initialization) to initialize the potential features of users and projects.

Because of the above problems, this paper combines the doctors' description document and patients' reviews to mine the potential relationship among doctors, patients, and services and proposes a Probabilistic Matrix Factorization integrated with the Convolutional Neural Networks (PMF-CNN). The main work is as follows:

1. CNN [32, 36] is used to automatically obtain the deep-seated features in the review information, and the influence of word order and context information on the extracted potential interest features of users can be considered simultaneously to generate a better potential feature representation than LDA model. Particularly when the user reviews matrix is sparse, the use of CNN is helpful to understand the review information in a profound way and generate a better potential model.
2. This paper proposes an initialization method of hidden layer representation of pretraining data through layer by layer unsupervised learning. We use the depth Stacked Denoising Autoencoders (SDAE) [37] to enter through the review information related to the value of doctors. The best initial value of doctors and patients review information can be obtained by row reconstruction, which can effectively improve the learning efficiency and performance of the matrix decomposition process.
3. We will test the proposed method on the MovieLens100k dataset and compare it with NCF, which is a de facto benchmark for deep learning recommendation system algorithms, to verify the effectiveness and accuracy of this method. At the same time, we will analyze the important parameters affecting the PMF-CNN recommendation performance. Experimental results show that the proposed algorithm is superior to other advanced algorithms.
4. This paper proposes a framework based on a deep learning model and probabilistic matrix decomposition model to integrate the relevant information of patients' reviews and doctors' professional knowledge and uses it to predict the patients' preference for the corresponding doctor and gives the specific modeling process. Based on the Haodf dataset, experiments are carried out to verify the performance of the proposed algorithm. Experimental results show that the proposed algorithm is superior to other advanced algorithms.

The paper is arranged as follows: Section 2 introduces the related work, especially in convolutional neural network; Section 3 describes the specific methods of this paper, mainly including the related theory and algorithm implementation; Section 4 is the experiment and analysis; Section 5 gives the summary and outlook.

## 2. Convolutional Neural Network (CNN)

### 2.1. The Structure of CNN

Convolutional Neural Network (CNN) is essentially a kind of nonprobabilistic model of multilayer perceptron [38, 39]. However, its architectural differences have significant practical consequences. Although CNN was originally developed for computer vision, its key ideas have been actively applied to information retrieval and natural language processing (NLP), such as search and retrieval, sentence modeling, and classification (traditional NLP tasks). CNN can make good use of the prior information of “spatiotemporal locality” [40] in the data. CNN extracts features from the original data dynamically in the hidden layer between the input layer and the output layer and then applies these features to the classification or fitting of the subsequent output layer. We use CNN to preliminarily explore how to solve the recommendation problem, which has achieved good results in doctor recommendation and demonstrated the feasibility of deep learning in the recommendation system. Sometimes, we want to predict an ordered set (such as a sentence sequence composed of words), such as the emotional tendency of prediction sentences (positive, negative, and neutral). We can find that most of the time, only a few words in a sentence provide useful information, while other words provide little or no information. For example, in the sentence “I am very happy today,” the word “happy” has provided enough information to show that the sentence expresses positive emotions. So, the key of the problem is how to select these words with large amount of information. This paper mainly uses CNN to extract this useful information automatically.

There are four layers in the whole CNN network.

- Input layer: it is a matrix in which the word vectors corresponding to the words in the sentence are arranged in turn (from top to bottom). If there are *n* words in the sentence and the dimension of the vector is *k*, then this matrix is *n* × *K*. The type of matrix can be static or dynamic. Static means that the word vector is fixed, while dynamic means that, in the process of model training, the word vector is also regarded as an optimization parameter. For the unknown word vector, it can be filled with 0 or random small positive number.
- Convolution layer: it obtains several feature maps through convolution operation. The size of convolution window is *h* × *d*, where *h* represents the number of vertical words and *d* represents the dimension of word vector. Through such a large convolution window, several feature maps with 1 column number will be obtained.
- Pooling layer: the pooling layer adopts the method of Max over time pooling. This method simply presents the maximum value from the previous one-dimensional feature map, which represents the most important signal. As you can see, this pooling method can solve the problem of variable length sentence input (because no matter how many values are in the feature map, only the maximum value needs to be extracted). Finally, the output of the pooling layer is the maximum value of each feature map, which is a one-dimensional vector.
- Full connection + softmax layer: the output of one-dimensional vector of pooling layer connects a softmax layer through full connection. The softmax layer can be set according to the needs of the task (usually reflecting the probability distribution on the final category).

**Figure 1:**
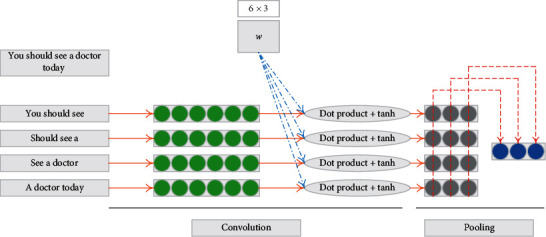
An example of sentence classification of CNN structure.

The core idea of CNN is to apply a nonlinear function to each word window (*k*-word window) of the input sentence. This nonlinear function is generally called convolution kernel (called filter in image processing), and this operation is called convolution operation. In this way, the window data of a *k*-word can be transformed into an *m*-dimensional vector through the application of filter. In the standard CNN structure, the convolution operation is generally connected with the pooling operation. The most common pooling operations include mean pooling and max pooling. In the field of natural language processing, the maximum pooling is widely used, because through the selecting the maximum value of the features generated by convolution operation is equivalent to obtaining the feature with the largest amount of information, that is, selecting the key words in a sentence.  Figure 1 gives a network model architecture of how to perform convolution and pooling operations. In this example, the input sentence is “you should see a doctor today,” where the word window *K* is selected as 3, so there are four window inputs, as shown in the leftmost figure. Suppose that each word is represented by a 2-dimensional vector, so the windows of three words can be represented by a 6-dimensional vector (as shown in the green part of the figure). Convolution operation is equivalent to applying convolution kernel *w* to each word window, in which *m* is selected as 3. Therefore, through convolution operation, a 6-dimensional vector will be converted into a 3-dimensional vector. In this example, the data of four windows will be converted into four 3-dimensional vectors, that is, the gray part of the graph. The final pooling operation is to select the maximum value of gray part column and finally generate a three-dimensional vector (blue part in the figure), which is the feature extracted by CNN.

#### 2.1.1. Convolution Operation

CNN subdivides the hidden layer according to the different operation and function and specifically divides it into convolution layer and pooling layer. These two hidden layers can directly learn the information features, so as to extract the features and avoid the extraction of artificial features.

CNN is a model based on multilayer perceptron, but the biggest problem of multilayer perceptron is that it is a fully connected network, so when the input is large, the weight will be especially large. This problem, on one hand, limits the maximum number of neurons that each layer can accommodate and, on the other hand, limits the number of layers of multilayer perceptron, that is, depth. In general, the input needs to be normalized, and the output of each neuron is also normalized under the action of the activation function; in addition, the absolute value of the effective parameters is generally less than 1. In the process of back propagation, multiple numbers less than 1 are multiplied to get smaller values. In other words, with the increase of depth, the residual from the back to the front will be smaller and smaller and even cannot help to update the weights, thus losing the training effect, making the parameters of the front edge layer tend to randomize, and the convolution operation will improve this problem very well.

Convolution operation exists in the convolution layer in the hidden layer. The convolution layer is directly connected with the input layer, and its number is generally consistent with the number of pooling layers. The main function is to enable the artificial neuron to respond to a part of the surrounding units within the coverage, extract the feature directly, and move multiple filters on the input matrix for feature learning. In terms of its structure, the biggest difference between it and the hidden layer in the general artificial neural network is that the connection mode of neurons is not all connected. The operation is similar to full connection, but the operation of full connection layer converts the input into a one-dimensional vector and then performs point multiplication on the one-dimensional vector, while convolution acts on a local area, that is to say, the sensing area of convolution layer is not. It is only part of the neurons in the upper layer. The local information will be integrated into the whole information in the later level. The biggest advantage of this connection is the sharp reduction of the number of weights. Different convolution kernels of different sizes act on the matrix in the middle and will convolute to get different characteristic graphs on the left and right.

The architecture parameter debugging method for sentence classification based on CNN is as follows: Each token of the input sequence is embedded into a 5-dimensional vector, so the input of the model is a 7 × 5 matrix. The first layer of the model is the convolution layer. There are six convolution kernels in the convolution layer: L1, L2, L3, L4, L5, and L6. Their sizes are 4 × 5, 3 × 5, and 2 × 5. Then, after convolution and activation function, six feature maps with sizes of 4, 4, 5, 5, 6, and 6 are obtained. Six feature maps were obtained by max pooling to obtain 6-dimensional feature vectors. Finally, the corresponding categories are predicted by softmax.

For the input sample *x*={*x*_1_, *x*_2_,…, *x*_*n*_}, if the word window length is *h*, then, for the input sample with length *N*, there are *N* − *h*+1 word windows. For ith word window, input *w*_*i*_ ∈ *R*^(*h* *d*)^, which is made up of the word vectors of *k* words in the window. If the convolution kernel is defined as *W* ∈ *R*^(*h* · *d*)×*m*^, then the convolution result *P*_*i*_ ∈ *R*^*m*^ can be defined as shown in the following formula:

(1) $$\begin{array}{c} p_{i}=f\left(w_{i}W+b\right), \end{array}$$

where *f* is a nonlinear function, such as sigmoid and tanh, while *b* is an offset.

Every time the window moves, it will perceive the local area covered in the window. The local area that it can perceive is called the perception field. Different window sizes can perceive the characteristics of different area sizes very well. Under the effect of different convolution kernels, we can get different size characteristic graphs. It is worth noting that, before and after each convolution kernel moves, the perceptual weights of each position in the window are the same. This way of weight sharing is also one of the characteristics of convolutional neural network, which helps to reduce the number of weights and the complexity of network model to a large extent.

#### 2.1.2. Pooling Operation

Pool layer is an important part of CNN, which can reduce the problem of overfitting. Its input is from the adjacent upper layer of the convolution layer. Its main function is to further sample the output characteristic map of the convolution layer, that is, to process the extracted characteristics of the convolution layer. The results of the pooling will participate in the subsequent training, so the pooling layer is also called the subsampling layer. Piczak give a good schematic visualization of a typical implementation process of convolution-pooling operation [41].

When the sampling window of 2 × 2 is used to downsample the information matrix of 4 × 4, the step size of the sampling window of the pooling layer is generally the size of the downsampling area. Compared with convolution operation with step size of 1, convolution layer will have overlapping of window areas, while pooling layer generally does not have overlapping of processing areas; that is to say, pooling operation is not continuous. Therefore, pooling operation is more effective in feature reduction when convolution operation and pooling operation are performed with windows of the same size, respectively. In addition, the width and height of the sampling area are not necessarily the same, and the pooled window is not easy to be too large, because the information loss may be serious at the same time of rapid dimensionality reduction.

The features extracted from the convolution layer are regarded as a matrix, and there are two common ways to deal with the matrix in the pooling layer: mean pooling and max pooling. Max pooling in this paper is to take the maximum value of a local area as the feature representative of the area after pooling, comprehensively select the most representative information, and choose the most relevant feature; that is to say, maximum pooling divides the input area into several nonoverlapping subareas, so that each subarea outputs its maximum value. After maximum pooling, the value is calculated according to the following formula:

(2) $$\begin{array}{c} \text{Value}=f\left(W_{pw}\times \max\left(\left(X_{p}\right)\right)+b\right), \end{array}$$

where Value represents gray block value, *W*_*pw*_ represents pooling weight matrix, *X*_*p*_ represents area matrix covered by pooling window, and *b* represents offset. *f* is the activation function.

### 2.2. Extraction Framework of Medical Consultation Sentence Relationship Based on Neural Network

**Figure 2:**
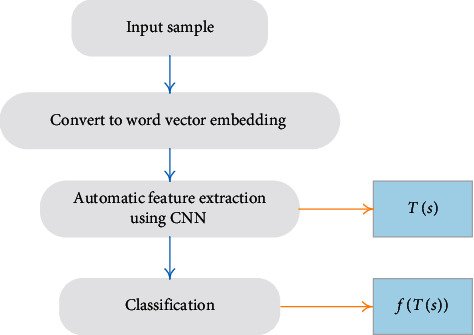
Medical consultation sentence relation extraction via neural networks.

The task of medical consultation sentence entity relation extraction can be described as follows: given a sentence *s*={*w*_1_, *w*_2_, *e*_1_,…, *w*_*j*_, *e*_2_,…, *w*_*n*_}, where *e*_1_ and *e*_2_ are entities, the mapping functions can be defined in the following formula:

(3) $$\begin{array}{c} f\left(T\left(s\right)\right)=\left\{\begin{array}{cc} =1, & \text{If there is a relationship between }e_{1}\text{ and }e_{2},\\ =-1, & \text{others}, \end{array}\right. \end{array}$$

where *T*(*s*) can be regarded as the feature extracted from a sentence containing entity pairs, and the mapping function *f* determines whether there is a relationship between the two entities, so *f* can be regarded as a classifier. In this paper, Bayesian classifier can be used as *f*. It can be seen from Figure 2 that this framework is consistent with our original definition of medical consultation sentence relation extraction in formula (3).

Compared with the traditional relationship extraction system based on machine learning algorithm, the proposed framework uses CNN neural network for automatic feature extraction on the basis of word vector, thus avoiding the process of manual feature extraction. In this paper, multichannel word vectors are introduced as the input of CNN, static and nonstatic pretraining word vectors are used, and the middle feature map is the sum of two feature maps.

## 3. Our Approach

In this section, we first describe the definition of the rating prediction task and the notation that we are going to use in this paper. Then, we introduce our CNN model to extract the sequential reviews of patients and doctors. At last, we utilize the sequential features as side information in the feature based collaborative filtering framework to make the final prediction.

### 3.1. Problem Definition

Given *N* patients (users) and *M* doctors (items), the rating *r*_*ij*_ is the rating given by *i*^th^ patient for *j*^th^ doctor. In the common real-world situations, patients usually rate on a fraction of doctors, not on the whole items. Therefore, those ratings entail a big and sparse matrix *R* ∈ *R*^*N*×*M*^. The goal of recommendation system is to make a prediction on the missing ratings. Based on that, we will know the preference of a patient on the doctors that he/she never rates and recommend high score items to him/her.

Table 1 summarizes the symbols used in the paper. In the next subsection, we will propose a CNN model to extract sequential features of users and items.

### 3.2. Doctor Recommendation Model

**Figure 3:**
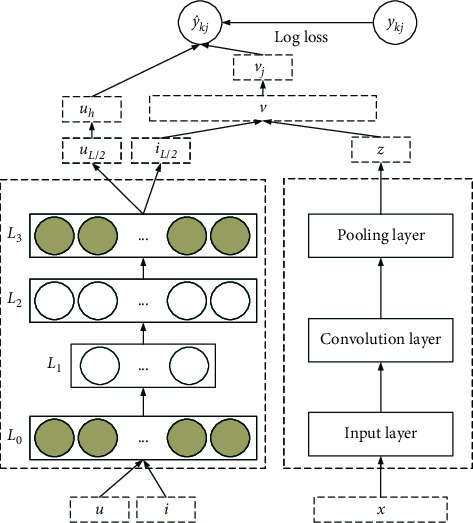
Doctor recommendation model based on probability matrix decomposition of hybrid neural network.

Figure 3 shows a detailed representation of the learning process of each component of the model. The dotted border on the left represents the preprocessing component of patients' reviews information, and the dotted border on the right represents the feature learning component of doctors' categories information. The input is a triple *u*, *i*, *x*, where *u* represents the user (patient) set, *i* represents the item (doctor) set, and *x* represents the reviews information set. Specifically, by learning the initialization parameters of users and items through SDAE, the optimal user characteristics and doctor characteristics are obtained; the potential feature vectors are obtained by Frequency–Inverse Document Frequency (TF-IDF) and learning reviews information through CNN network. Then, by fusing the review feature, the feature of the reasonable doctor ranking is obtained and the score of the doctor is predicted. The following is a detailed introduction to the learning process of each component of the model in Figure 3.

Table 2 summarizes our baseline PMF-CNN model.

ReLU (corrected linear unit) [42] is an activation function commonly used in deep neural networks. For patients' reviews documents, a simple baseline is to select the nouns with the highest frequency, i.e., appearing in as much reviews as possible. The common approach to extract feature is the term Frequency–Inverse Document Frequency (TF-IDF) as

(4) $$\begin{array}{c} \text{TF}-\text{IDF}\left(x\right)=\text{TF}\left(x\right)\times \text{IDF}\left(x\right)=\left(\log\frac{N}{N\left(x\right)}\right)\times \left(\log\frac{N\left(x\right)}{N\left(x\right)+1}+1\right), \end{array}$$

where *N* is the quantity of whole text data and *N*(*x*) is the quantity of text data in Table 2.

Probabilistic Matrix Factorization (PMF) is a classic collaborative filtering method to solve this problem [43, 44]. It aims to find a *K* dimensional low rank matrix $\hat{R}\in R^{N\times M}$ where $\hat{R}=UV^{T}$ with *U* ∈ *R*^*N*×*K*^ and *V* ∈ *R*^*M*×*K*^ are two matrices of rank *K* encoding a dense representation of the patients and doctors with

(5) $$\begin{array}{c} \underset{U,V}{\arg\min}\sum_{\left(i,j\right)\in K\left(R\right)}{\left(r_{ij}-{\overset{⟶}{u}}_{i}^{T}{\overset{⟶}{v}}_{j}\right)}^{2}+\lambda \left({\left\|{\overset{⟶}{u}}_{i}\right\|}_{\text{Fro}}^{2}+{\left\|{\overset{⟶}{v}}_{j}\right\|}_{\text{Fro}}^{2}\right), \end{array}$$

where *K*(*R*) is the set of indices of known ratings, ${\overset{⟶}{u}}_{i}$ and ${\overset{⟶}{v}}_{j}$ are the corresponding line vectors of *U* and *V*, *λ* is the coefficient that controls the influence of L2 regularization, and ‖·‖_Fro_ is the Frobenius norm.

In Algorithm 1, the pseudocode of PMF-CNN algorithm is given, including training, testing, and prediction.

To train two networks simultaneously using a single loss function in Figure 3, this paper combines the outputs of both networks by concatenation. From here, the interaction of patients' review features with doctor features is done via a PMF in which the details are not provided. However, the goal of the PMF is to capture second order interactions between patients and doctors. The PMF-CNN loss function including PFM:

(6) $$\begin{array}{c} \text{PMF}-\text{CNN}=\hat{\beta_{0}}+\sum_{i=1}^{\left|\hat{z}\right|}\hat{w_{i}}\hat{z_{i}}+\sum_{i=1}^{\left|\hat{z}\right|}\sum_{j=i+1}^{\left|\hat{z}\right|}\langle \hat{v_{i}}|,|\hat{v_{j}}|\hat{z_{i}}|\hat{z_{j}}\rangle , \end{array}$$

where ${\hat{\beta }}_{0}$ is the global bias, ${\hat{w}}_{i}$ models strength of *i*^th^ variable in $\hat{z}$, and $\langle {\hat{v}}_{i},{\hat{v}}_{j}\rangle$ is the 2^nd^ order interaction.

## 4. Experimental Study

### 4.1. Data Collection

As a new mode of public health service, online medical website has developed rapidly in China. Haodf.com (referred to as Haodf) only includes doctors from public hospitals, but not from private hospitals. When doctors register and open medical services, they need to submit professional title certificates, qualification certificates, and so on. Haodf also has special departments to verify the authenticity of doctors' information, so doctors are all true. Haodf collected 3856035 real votes, comments, and thank-you letters from 194.65 million patients in 605066 doctors' outpatient clinics in 9823 public hospitals across the country. They share their experience of diagnosis and treatment or their subjective feedback on the treatment effect of the doctor, their psychological care for the patient, and their attitude towards coping with the disease together with the patients, which is a good help for more patients to identify their own diseases: which doctor should the patient look for and which doctor is the best that the patients should trust and entrust their own recovery or even life. Relying on the Internet, intellectualization is the inevitable trend of online medical consultation website. Through intellectualization, Haodf can provide more accurate medical and health services, so as to help the construction and development of public health.

The MovieLens100k 1 dataset was used as benchmark experiment dataset. 80% of the dataset is randomly divided as training data and the remaining 20% is used as test data. From 21 July to 20 August 2018, approximately 2 million doctor patient interaction data were obtained from Haodf through web crawler. The data with personal web pages on the online platform of doctors were collected and analyzed from the aspects of medical life, patient visits, and patient satisfaction. The doctors and hospital resources provided by Haodf online platform are mainly public tertiary hospitals, and the professional titles of doctors are mainly intermediate and deputy senior. Only one-third of the doctors with open personal homepage have high activity, and the patients' satisfaction score of the platform is high. The analysis tool of review and ontology used in this paper has two parts: Jieba Chinese word segmentation 2 and Chinese emotional vocabulary ontology of DUTIR 3, respectively.

The density metric [45] in Table 3, which means that how much elements are rated, is calculated according to the following equation:

(7) $$\begin{array}{c} \text{Density}=100\times \frac{\#\text{ available ratings}}{\#\text{ all possible ratings}}=100\times \frac{\#\text{ available ratings}}{\#\text{ users}\times \#\text{ items}}. \end{array}$$

### 4.2. Evaluation Criteria

When we evaluate a recommendation system, it is not possible to evaluate only one user's recommendation list and corresponding results, but the entire test set of users and their recommendation list results. The Average Precision (AP) reflects that the indicators are somewhat similar to the concept of recall, except that it is the sequentially sensitive recall. The AP for *u* is defined as

(8) $$\begin{array}{c} AP=\frac{1}{\left|I_{u}^{te}\right|}\sum_{i\in I_{u}^{te}}\frac{\sum_{j\in I_{u}^{te}}\delta \left(p_{uj}≺p_{ui}\right)+1}{p_{ui}}, \end{array}$$

where *p*_*ui*_ indicates the sort position of the item *i* in the recommendation list. *p*_*uj*_≺*p*_*ui*_ indicates that the item *j* is in front of the item *i* in the sort list for user *u*.

In this paper, the Mean Average Precision (MAP) and the Normalized Discounted Cumulative Gain (NDCG) are used as evaluation indexes to evaluate the performance of recommendation algorithm 1. The MAP indicates the proportion that the first *n* items recommended can hit the user's actual preference, while the NDCG indicates the ranking quality of the recommendation list.

The MAP is divided into two parts. First, the average accuracy of sorting is calculated, and then the average accuracy of the whole is calculated. The MAP is just the average of all users' AP. The MAP for *u* is defined as

(9) $$\begin{array}{c} \text{MAP}@u=\frac{\sum_{u\in U^{te}}\text{AP}@u}{\left|U^{te}\right|}. \end{array}$$

Then, the evaluation scores of different users' recommendation lists need to be normalized by the NDCG. The value of the NDCG is between (0,1]. The NDCG@*u* for *u* is defined as

(10) $$\begin{array}{c} \text{NDCG}@u=\frac{\sum_{i=1}^{p}\left(2^{{\text{rel}}_{i}}-1/\log_{2}\left(i+1\right)\right)@u}{\left|U^{\text{te}}\right|}, \end{array}$$

where rel_*i*_ indicates the relevance of the recommendation results in position *i* and *u* indicates the size of the recommendation list to be examined. Then,

(11) $$\begin{array}{c} \text{NDCG}@u=\frac{\sum_{u\in U^{te}}{\text{NDCG}}_{u}@u}{\left|U^{te}\right|}. \end{array}$$

### 4.3. Experimental Process and Analysis

Among the evaluation criteria, the performance of the model under different parameters is evaluated many times. In the experiment, MAP, NDCG@3, NDCG@5, NDCG@10, and NDCG@20 were selected as evaluation indexes. We compared the evaluation indexes of MAP and NDCG between PMF-CNN and NCF [28] in Table 4.

Table 4 shows the MAP and NDCG results of PMF-CNN and NCF in different dimensions. Obviously, PMF-CNN recommendation with content information and adaptive sampling strategy is better than NCF, which shows that content information and convolution sampling strategy have strong feature extraction ability and generalization ability. The results also prove that the improved model proposed in this paper has good feasibility and validity in online doctor recommendation system.

The training execution time of the PMF-CNN and NFC in Table 5.

Many big data technologies are used in the field of natural language processing [46]. At the same time, deep learning is more and more widely used in the field of big data such as syntax analysis, text classification, and sentiment analysis. In this paper, the diagnosis and treatment data analysis uses big data processing technology. We analyze the effect of PMF-CNN on doctors' recommendation of ophthalmology, which is a common medical category in Haodf website. The satisfaction degree of patients with different degree of education to the doctor's diagnosis and treatment results is different. Patients with primary school education had the highest satisfaction with diagnosis and treatment results, while patients with bachelor's degree or above had the lowest satisfaction. We selected 5 diseases (Cataract, Dacryocystitis, Conjunctivitis, Keratitis, and Myopia) from ophthalmology for a case study.  Table 6 shows the top 5 recommendation results of ophthalmologists in Shanghai. There are 2479 ophthalmologists in Haodf website. We found that most of the recommended doctors were affiliated to a famous eye hospital, such as Fudan University Affiliated Ophthalm Otolaryngology Hospital; this powerful specialized hospital has skilled doctors. Our recommendation results were validated in Table 6.

## 5. Conclusions

In this paper, a hybrid recommendation algorithm (PMF-CNN) based on deep learning is proposed for doctor recommendation, and an automatic depth encoder is used to learn the initial value of potential eigenvectors of patients' reviews and doctors' professional knowledge in the process of matrix decomposition. PMF-CNN model uses convolutional neural network to learn the context features of review information, so as to extract more accurate feature representation to realize the modeling of review information. For the modeling of interaction between patients' reviews and doctors' professional knowledge in the matrix decomposition model, the best initial value of potential eigenvectors of patients' reviews and doctors' professional knowledge is learned by using the noise reduction automatic encoder to effectively avoid falling into the local optimal solution in the process of matrix decomposition. Finally, the matrix decomposition technology is used to integrate the above two kinds of modeling to provide the patient recommendation service. The verification results on the Haodf dataset show that PMF-CNN is obviously superior to comparative recommendation algorithm.

However, PMF-CNN has the problem of cold start; that is, it can only recommend on the historical doctors and cannot evaluate other new doctors. Therefore, the following research will consider adding features of medical consultation category and patients' reviews to get the representation of category and reviews, so as to solve the problem of cold start and improve the accuracy of recommendation. In the future work, it will be an interesting direction to integrate multiple context information based on deep learning framework. It is also a should be adaptive on the basis of a results-driven approach in the interface [47].

## Figures and Tables

**Algorithm 1:**
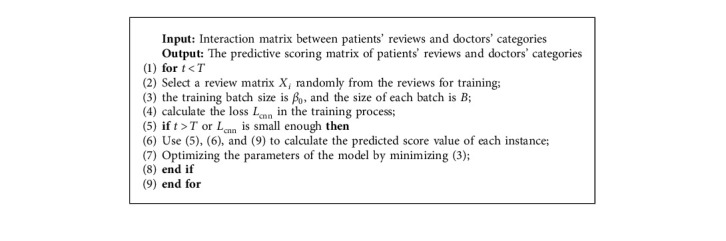
PMF-CNN algorithm.

**Table 1:** Summary of notations.

Notation	Description
*N*	Number of patients
*M*	Number of doctors
*K*	Dimension of latent factors
*D*	Dimension of sequential features
*R* ∈ *R*^*N*×*M*^	Rating matrix
*U* ∈ *R*^*N*×*K*^	Latent factors of patients
*V* ∈ *R*^*M*×*K*^	Latent factors of doctors
*X* ∈ *R*^*N*×*D*^	Sequential features of patients
*Y* ∈ *R*^*M*×*D*^	Sequential features of doctors

**Table 2:** PMF-CNN baseline architecture.

Parameter name	Parameter setting	Description
**CNN layer**	2	The number of CNN layers
*HiddenSize*	16	The number of hidden layers
*Filters*	2	The number of filters
*KernelSize*	4	The number of kernels
*Strides*	2	The number of strides
*Activation*	ReLU	Activation function
**Max pooling**	1	The number of max poolings
**Flattened**	1	Flattened convolution
**Fully connected**	1	Fully connected layer
*Dropout*%	0.10	Discard rate
*λ*	0.01	Regularization coefficient

**Table 3:** Statistics of the two datasets used in this paper.

Dataset	Items	Users	Ratings	Density (%)	User features	Items features
MovieLens 100k	1,682	943	100,000	6.30	Age, gender, and occupation	Genres and year
Haodf	12,000	58,000	220,000	4.10	Doctors' positional titles	State of an illness

**Table 4:** Experimental evaluation of MAP and NDCG in different dimensions of *u*, using two metrics.

**NCF**	*u* = 10	*u* = 20	*u* = 30	*u* = 40	*u* = 50
MAP	0.1069	0.1039	0.0886	0.0875	0.0869
NDCG@3	0.3788	0.3539	0.3393	0.3049	0.2489
NDCG@5	0.4291	0.4151	0.3698	0.3611	0.2979
NDCG@10	0.4630	0.4456	0.4118	0.4020	0.3161
NDCG@20	0.4571	0.4321	0.4159	0.4127	0.3412

**PMF-CNN**	*u* = 10	*u* = 20	*u* = 30	*u* = 40	*u* = 50
MAP	0.1278	0.1234	0.1072	0.1068	0.1021
NDCG@3	0.4633	0.4339	0.4160	0.3788	0.3042
NDCG@5	0.5072	0.4748	0.4535	0.4152	0.3651
NDCG@10	0.5451	0.5098	0.4908	0.4461	0.3864
NDCG@20	0.5285	0.5079	0.4952	0.4324	0.4178

**Table 5:** Results—training execution time comparisons.

	Embedding	Training time
**CNN-PMF**	**100 dimensions**	**0 hrs 17 min 45 s**
NFC	100 dimensions	1 hr 49 min 52 s

**Table 6:** A case study of doctor recommendation in ophthalmology.

Diseases	Doctors
Cataract	Xingtao Zhou, Yinghong Ji, You Li, Luo Yi, and Xiaoying Wang
Dacryocystitis	Yan Wang, Lan Gong, Kaiming Su, Jing Li, and Yifei Yuan
Conjunctivitis	Wenqing Zhu, Jiaxu Hong, Hong Liu, Haifeng Qin, and Xinrong Zhou
Keratitis	Zhensheng Gu, Yanjun Hua, Jiaxu Shen, Chunyi Shao, and Peiquan Zhao
Myopia	Peijun Yao, Meiyan Li, Jing Zhao, Jinghui Dai, and Jifang Liang

## Data Availability

All the data used in this paper are obtained by Python crawler programming from the HaodF (https://haodf.com), one of the most popular online medical communities in China.
